# The COVID-19 Israeli tapestry: the intersectionality health equity challenge

**DOI:** 10.1186/s13584-023-00567-8

**Published:** 2023-04-25

**Authors:** Efrat Shadmi, Mohammad Khatib, Sivan Spitzer

**Affiliations:** 1 Faculty of Social Welfare and Health Sciences, University of Haifa, Haifa, Israel; 2 Zefat Academic College, Zefat, Israel; 3 Azrieli Faculty of Medicine, Bar-Ilan University, Zefat, Israel

**Keywords:** COVID-19, Health equity, Intersectionality, Minority populations

## Abstract

**Background:**

COVID-19 is disproportionately affecting disadvantaged populations, with greater representation and worse outcomes in low socioeconomic and minority populations, and in persons from marginalized groups. General health care system approaches to inequity reduction (i.e., the minimization of differences in health and health care which are considered unfair or unjust), address the major social determinants of health, such as low income, ethnic affiliation or remote place of residents. Yet, to effectively reduce inequity there is a need for a multifactorial consideration of the aspects that intersect and generate significant barriers to effective care that can address the unique situations that people face due to their gender, ethnicity and socioeconomic situation.

**Main body:**

To address the health equity challenges of diverse population groups in Israel, we propose to adopt an intersectional approach, allowing to better identify the needs and then better tailor the infection prevention and control modalities to those who need them the most. We focus on the two main ethnic – cultural—religious minority groups, that of Arab Palestinian citizens of Israel and Jewish ultra-orthodox (Haredi) communities. Additionally, we address the unique needs of persons with severe mental illness who often experience an intersection of clinical and sociodemographic risks.

**Conclusions:**

This perspective highlights the need for responses to COVID-19, and future pandemic or global disasters, that adopt the unique lens of intersectionality and equity. This requires that the government and health system create multiple messages, interventions and policies which ensure a person and community tailored approach to meet the needs of persons from diverse linguistic, ethnic, religious, socioeconomic and cultural backgrounds. Under-investment in intersectional responses will lead to widening of gaps and a disproportionate disease and mortality burden on societies’ most vulnerable groups.

## Background

It has been widely established that the COVID-19 pandemic affected population groups differently. Ethnic minorities as well as geographic and social peripheral populations in countries across the globe experienced significantly higher rates of morbidity and mortality [1–3]. The mounting evidence of these differences served as a powerful looking glass for highlighting existing health and health care inequities by which structural societal differences create unfair and unjust access and quality of care [4]. These multifactorial inequities that have exacerbated COVID-19 health and health care inequities, stress not only differences between populations, but the intersections between multiple social identities and social determinants [5].

The intersectional approach does not view health inequity as binary, but rather considers multiple interactive axes of relations such as ethnicity and gender or socioeconomic status and age [6, 7]. This multifactorial view can highlight the complexities of addressing multiple axes of identities when implementing policies and interventions. As the COVID-19 pandemic progressed, it became clear that a one size fits all approach focusing on a single determinant of inequity may be missing the mark. For example, an intersectional lens enabled researchers to demonstrate that gender interacts with people's social backgrounds to shape their vaccine hesitancy in a complex way, depending on the dimensions of socioeconomic status, showcasing that poverty and being employed were only associated with women's, but not with men’s, vaccine hesitancy. [8, 9]

Israel, along with many other countries around the globe, faced immense societal and health challenges during the COVID-19 pandemic [10]. The government’s response to the pandemic of social distancing, lockdowns, vaccinations roll out, and public communication evolved over time, and included a broad range of stakeholders, policies, and interventions [11]. The uniqueness of Israel’s tapestry, the multiple ethnic, cultural, religious, and even health identities people hold create de-facto a myriad of intersectional axes. We present three case studies on COVID-19 in Israel using a health equity intersectional lens. We examined inequities related to two types of distinct ethnic – cultural—religious minority groups, that of Arab Palestinian citizens of Israel and Jewish ultra-orthodox (Haredi) communities. Additionally, we present a focus on a sector of the population that experiences significant inequities in health and healthcare due to intersections of multiple factors, and is, unfortunately, often overlooked—persons with severe mental illness.

## Main text

### The Arab palestinian citizens of Israel

Arab Palestinian citizens of Israel constitute nearly 21% of the Israeli population [12]. Their social and structural disadvantages, when compared with that of the Jewish population, are expressed in multiple axes including education, health, socioeconomic status, unemployment rates as well as political and social marginalization. [13] Most Arab municipalities are ranked in the four lowest socio-economic clusters (95% vs.17% of Jewish municipalities) [14]. Forty-five percent of Arab families, 58% of Arab children, and 56% of the elderly population live below the poverty line, in comparison to the national average poverty rate of 18% and 30% of children [15]. Health disparities are apparent in indicators such as life expectancy (80 years in the Arab compared to 84 in the Jewish population), general and infant mortality (4.7/1000 live births compared to 1.6 in the Jewish population) [12], and chronic morbidity, as well as the availability and access to various health services, despite universal health coverage mandated by a National Health Insurance Law [16].

These inequities are an expression and a result of structural and social determinants reflected in the distribution of resources, budgets, infrastructures, and services. Yet, the multifactorial inequities experienced by the Arab Palestinian citizens of Israel are not just in comparison to the Jewish population; within the Arab population, there are also disparities according to the region of residence. More than 70% of the Arab population lives in the northern and southern periphery of the country. Those residing in Israel’s southern periphery are younger (49% aged 0–14 years), but suffer from lower health, education, and welfare services, a lack of physical infrastructure and employment in comparison to Arabs living in the north or center of the country [17].

Arab society seemed to be impacted by the COVID-19 pandemic a bit later than the Jewish society. At the end of March 2020, Ministry of Health data showed few cases of COVID-19 in the Arab population (significantly lower than in the general population). These surprising data raised an urgent question regarding access to COVID-19 testing in Arab localities. In early March 2020, Israel faced a shortage of tests and COVID-19 tests were only conducted by emergency services in the community for people who experienced symptoms. Testing centers were established mainly in the big cities and were less accessible to the Arab population, most of whom live in villages. As a result, the testing process was extremely slow in the Jewish local authorities, but even slower in Arab local authorities. This situation led to cooperation and coordination among medical, social, and political leadership of the Arab population to encourage the Arab population to perform tests, and later, when vaccines were made universally available, also to get vaccinated [18].

By the end of the first wave of the pandemic in Israel (June 2020), only 2,060 confirmed cases and 11 deaths were registered in the Arab society (compared to 21,285 and 297 in the Jewish society respectively) [19]. Yet, a sharp increase in morbidity and mortality was observed with the outbreak of the second and third waves (end of 2020) [20]. However, the incidence of COVID-19 in Arab society was not uniform. Clear differences were noted between regions (southern vs. northern district) and towns. Gender differences were also observed with more women infected than men. By the end of the third wave (mid-2021), the mortality rate in Arab society was extremely high, even though morbidity was not significantly higher than that in the general society. Interestingly, when examining the data on vaccinations, more than 50% of the adult population in Israel was vaccinated by the end of 2020 and the first half of 2021 (4.82 million with the first dose and 3.5 million in the second dose) [21]. However, despite the country’s COVID-19 vaccination campaign, data from the Ministry of Health showed low response rates among the Arab population. The Arab population’s low levels of trust in the country’s national systems probably contributed to the relatively slow uptake of vaccinations.

The variability in the responsiveness to the testing, social isolation and eventually vaccination requests and requirements throughout the pandemic was related to the intersections between the local and national authorities and leaders. During the first wave, there were significant delays in getting the pandemic mitigation efforts clearly communicated to the Arab population. With the distribution of the required information in Arabic and its wide accessibility to the public, messages were more clearly and culturaly appropriately conveyed. The religious leadership and the Islamic Council played an important role in this. In addition, Arab doctors and medical staff members, as well as civil society organizations, and the public media were mobilized to increase awareness in the community. On the other hand, most Arab local authorities, including the Bedouin settlements in the south were not prepared to function in the reality of a continuous state of emergency. To address these challenges task forces were established in Northern and Southern Arab localities, and a representative was appointed (Mr. Aiman Saif) to lead the coordinating efforts of the Ministry of Internal Affairs, the Ministry of Health, and the national COVID-19 headquarters as they related to response efforts in Arab localities [22].

During the second COVID-19 wave, and the expansion of infections in Arab localities (mainly related to large gatherings in weddings and commercial centers) coordination efforts with local leaders were renewed and tailored solutions were delivered, to provide food packages to people in isolation and emphasize religious and community leaders calls for adhering to mitigation and control requirements. Nonetheless, the financial strains and political mistrusts surfaced, highlighting the implications of lockdowns on already strained and unstable economic and social conditions [23]. Additionally, social and cultural factors played an important role in vaccine uptake. For example, a survey on willingness to get vaccinated revealed ethnic group, gender and education differences, with the highest rates of refusal to get vaccinated reported by Arab women [24]. Overall, throughout the pandemic, the intersection between risk factors (e.g., excess smoking and diabetes rates coupled with low socio-economic status and slower vaccine uptake among persons from Arab localities) are considered to have contributed to the overall higher excess mortality in the Arab population relative to Jews and Others, especially in the third wave an amongst older adults. [20]

### The Jewish ultra-orthodox (haredi) communities

Ultra-Orthodox Jews currently constitute 13% of the total Israeli population [25]. Characterized by their strict following of the Jewish law and adherence to religious leadership, this community often avoids modernism, secluding themselves from Jewish secular culture [26]. Additionally, Jewish ultra-orthodox society is characterized by social conformity, strong community ties and support. This population is characterized by low socioeconomic status and economic hardships related to men’s full-time study of religious texts, women’s employment in low-paid occupations and having large families (an average of 6.9 children per family). [25, 27]

The first wave of the COVID-19 pandemic hit the Ultra-Orthodox population hard. This population suffered from significantly higher incidence of infection in comparison to that of the general Jewish population [21]. Despite being a minority, Ultra-Orthodox Jews accounted for more than one-third of Israel’s confirmed COVID-19 cases, 60–70% of COVID related hospitalizations, with mortality being twice as high in comparison to the general Jewish population [28]. In April 2020, Israeli police sealed off and declared the predominantly ultra-orthodox city of Bnei Brak, the city with the highest population density in Israel, a restricted zone due to 38 percent of the city’s residents being infected with coronavirus. This significant incidence of infection may not come as a surprise, as it reflects the intersectional interplay of identities of the ultra-orthodox population with social and structural determinants. The low socio-economic status of ultra-orthodox families has large families living in small and overcrowded homes. Additionally, the importance of community and communal religious actions, such as prayer, resulted in widespread disregard and even opposition to government policies [29, 30]. The lack of adapting policies and interventions to the importance of community practice came forward in higher rates of vaccination hesitancy among the ultra-orthodox population, despite national coverage and rollout. A population study that used Israeli National COVID-19 data (until 10 May 2021) found the lowest vaccination rates in areas predominantly populated by Ultra-Orthodox Jews. Importantly, Ultra-Orthodox women had significantly lower adjusted vaccination rates than all other population groups [31]. This may be a result of the relatively slow response of Israel’s Ministry of Health in creating tailored communication for the ultra-Orthodox population as well as working with religious leadership to mandate, for example, social distancing [32]. The involvement of religious leadership increased compliance to public health regulations significantly.

It is important to note that much of the COVID-19 discourse has lumped Ultra-Orthodox communities and their members together overlooking the heterogeneity that exists within this population group. The different groups cover a very wide range of ideological positions and identities towards health, ranging from radical anti-Zionist to the more mainstream [28]. Effective mitigation and containment efforts included a combination of recruitment of support from religious leaders and charity organizations, door-to-door provision of essential services and good, including food and drugs, increased testing and isolation, and stricter physical distancing measures in severe outbreak areas [32]. While most religious leaders and communities, following work of the Ministry of Health, adhered to COVID-19 recommendations, some groups did not. Interestingly, criticism from within the ultra-Orthodox community was directed towards religious leaders who advocated violating the MOH guidelines. This new voice may indicate that for some ultra-orthodox people, personal perception of health may outweigh obedience to religious authorities. [33]

### Persons with severe mental illness

Persons with severe mental illness (SMI), especially those diagnosed with schizophrenia, are at increased risk for COVID-19-related severe morbidity and mortality [34, 35]. Persons with SMIs may be considered a high-risk group for severe COVID effects due to their multiple intersectional factors, including poor socioeconomic status, poor living conditions, difficulty in accessing and using health services and accompanying physical comorbidities [36]. Indeed, a recent meta-analysis has shown that mental disorders were associated with significantly higher COVID-19 illness severity and mortality rates. Susceptibility to contracting COVID-19 was associated with pre-existing mood disorders, anxiety, and attention-deficit hyperactivity disorder (ADHD); illness severity was associated with mood disorders; and mortality was associated with schizophrenia [37].

Evidence from Israel presents a similar picture, showing that persons with schizophrenia were more likely to suffer from COVID-19 morbidity and mortality compared to age and gender matched controls [38]. Moreover, findings from a large-scale study of health records of over 50 thousand Israeli patients with Schizophrenia, indicate that they were under-vaccinated for COVID-19 compared to the rest of the population. This gap between persons with and without schizophrenia was most pronounced in people aged 60 and above, where risk factors converge [39]. Follow-up on the uptake of the booster (third) vaccine showed that gaps in vaccination were attenuated, yet still remained (75% in the schizophrenia group vs. 78% in controls) [40]. Another national study linked data on past psychiatric hospitalization and COVID-19 testing, infection, hospitalization, mortality, and vaccinations, between March 1st 2020 and March 31st 2021. That study showed that persons with SMIs were less likely to be tested for COVID-19 and that among those infected, risks for COVID-19 hospitalization and mortality were higher than the total population, adjusting for age, sex, vaccination status and physical comorbidities. National vaccination rates for persons with a former psychiatric hospitalization were 60% compared to 75% for the totalpopulation. [41]

These gaps should be viewed in reference to the extensive local distribution of vaccines, and high accessibility of vaccinations in Israel. Nonetheless, for persons with SMIs vaccine uptake is complicated by several factors. First, vaccination hesitancy is generally higher, partly related to the underlying mental and cognitive conditions [42]. Second, persons with SMIs experience more obstacles to receipt of preventive care in general, as they may find that appointment scheduling and navigating the healthcare system to be difficult. Finally, indirect costs, related to travel time and cost, or time off work, may disproportionately affect persons with SMIs who are overrepresented in low socioeconomic groups. For these reasons, in January 2021 all patients hospitalized in psychiatric hospitals in Israel were vaccinated. Given the intersectionality of risks present in the SMI population, including low socioeconomic status, higher risk of severe outcomes if infected, due to comorbid physical conditions, and low vaccine uptake, future outbreak responses should consider prioritizing persons with mental illness, similar to the precedence given to healthcare workers and older adults.

## Discussion

Looking at COVID-19 in Israel through the unique lens of intersectionality and equity highlights the need for responses that addresses multiple axes. This requires that the health system creates multiple messages and intervention in which different intersectional identities are addressed.

COVID-19 revealed dimensions of inequity and their effect on Arab society and on Ultra-orthodox Jewish populations, in roll-outs of testing operations and vaccination take-up. These differences are rooted within the larger social and political context of the Arab Palestinian society within Israel. Arabs in Israel are suffering from underrepresentation in the institutional systems at the state level of decision-making processes. Thus, despite a National Health Insurance Law and mandated universal coverage, fewer services are provided in many areas [43]. In addition, The Arab minority, as a geographical and social periphery, has many needs that characterize the peripheral areas, such as lack of and low access to social and health care services, lower levels of employment, and cultural and linguistic gaps [44].

The Ultra-orthodox Jewish minority is also affected by the complexities of personal and societal risks, related to the areas of residence, the socioeconomic level and the cultural and religious characteristics which characterize many aspects of daily life, especially as they relate to social gatherings and their confrontation with social-isolation requirements. These intersectional needs, coupled with additional layers of internal and external politics, mistrust in officials, and lack of culturally tailored messages and services (especially in the early stages of the pandemic), can significantly affect the longer-term response to additional COVID-19 waves and potential subsequent breakouts and pandemics, and call for an in-depth account of the challenges and unique responses needed in regards to minority population groups.

COVID-19 caught the Israeli health system at a time in which it was supposed to be better than ever prepared to deal with the manifestation of viral outbreaks in persons with SMIs. About five years after the initiation of the mental health services insurance reform [45], which has transferred responsibility for mental health services to the Health Funds (non-for-profit insurers and providers pf services), enabling integration of all healthcare aspects related to physical and mental illnesses. Yet, anecdotal evidence presents the incompatibility of the overcrowded mental health hospitals to deal with the need to isolate persons and treat persons with SMIs who were COVID-19 positive, in mental health hospitals, separate from the general hospitals’, mostly fully prepared and operated, COVID-19 units. With patient discharge committees’ reduced functionality during COVID-19, higher rates of distress among persons with SMI due to the effects of social isolation and the lower accessibility of outpatient and community services, the strain on the mental health system has unsurprisingly increased. Thus, the COVID-19 experience points to the need to integrate the testing, vaccination, and treatment responses (for mild COVID-19 cases) for persons with SMIs in specifically tailored primary care and community mental health clinics.

Some lessons can be learned from the more advanced stages of the pandemic control efforts, which were more closely tailored to meet the intersection of needs of each of the minority groups addressed in this perspective. Israel’s strong primary care system, with community level accessibility to primary care physician, nursing and pharmacy services throughout the country, has played an important role in the relative equitable distribution of testing, treatment and vaccination services to all its residents. The fact that at the primary care level, care is often delivered by providers who are from the same cultural and linguistic background of their patients, also significantly contributed to the ability to provide a culturally tailored COVID-19 response. Additionally, the establishment of culturally tailored task forces which spearheaded the vaccination campaigns in Israeli Arab Palestinians and ultra-Orthodox Jewish populations has played an important role in addressing the cultural as well as logistic gaps and resulted in an increase vaccine uptake [46]. Similarly, increasing vaccination accessibility to persons with SMIs, especially for hospitalized patients, exemplifies the need to generate new structures and processes that tailor care to persons especially prone to be affected by the *inverse care law*, those most in need but who receive least of the care [47]. Further efforts aimed at populations with SMIs should further consider community resources as well as the advanced mental health rehabilitation scheme as potential facilitators to the delivery of preventive care, including immunizations.

This perspective is limited by taking an intersectional approach to examining the strategies to meet some of the main health equity challenges related to Israel’s COVID-19 response in its main Arab and Ultra-orthodox minority populations, and a unique intersectional view of persons with SMIs. Other populations, of low socioeconomic status, or other minority groups, have also been disproportionally affected by the pandemic. Beyond the scope of this specific perspective are also the inequitable larger societal local and global effects of the pandemic, including the loss of health insurance, jobs and homes, with increased risk for mental and physical morbidity long-term consequences, especially for disadvantaged populations. Detrimental consequences to human rights protection and the need for uniquely tailored solutions for vulnerable populations, including refugees, undocumented residents, and persons in prostitution have been called for, and at least in part addressed by joint efforts of NGOs and the Ministry of Health [10].

## Conclusions

Considering the case examples highlighted here, coupled with international lessons from efforts to reduce the inequitable effects of COVID-19 and its responses [48], the call for an intersectional lens in pandemic preparedness is highlighted. A comprehensive response that takes into consideration the complexities involved in ensuring access to high quality, ethnically, religiously and culturally tailored care need to be systematically and continuously implemented. Despite its reputation as a world leader in availability of health technology and data, Israel lacks a comprehensive, ongoing systematic approach that tracks gaps in health and health care across a broad range of indicators and populations. Some progress has been made in this regard by Wilf-Miron and colleagues [49] who conducted a consensus building process to develop a national set of health equity indicators. The process concluded with the identification of ten leading indicators, including: diabetes care, childhood obesity, adult obesity, distribution of healthcare personnel, fatal childhood injuries, cigarette smoking, infant mortality, ability to afford care, access to psychotherapy and distribution of hospital beds. These indicators present an important cornerstone of inequity measurement as the foundation to elimination of healthcare and health inequities. Yet, they are limited to mainly processes, rather than outcomes of care, manifest a healthcare centric, rather than a person-centered approach (e.g., do not include person reported outcomes), and most unfortunately, have not been adopted yet. Other approaches to health inequity monitoring have suggested a more comprehensive and standardized process, such as the World Health Organization Inequality Monitor (https://www.who.int/data/inequality-monitor). As in Israel, healthcare is still perceived as a basic right, establishing a standardized national assessment scheme can serve the larger societal goals of health inequity monitoring, as a foundation to subsequent efforts to eliminate unwarranted gaps in health and health care. Moreover, joint national efforts to track and eliminate inequities, led by the Ministry of Health and the four Health Funds are key to ensuring that future calamities do not disproportionately affect those who are most vulnerable. The foundations of a strong public health and primary care system, coupled with low- and high-tech solutions should be further developed to achieve a universal, equitable response to the ongoing short and long-term COVID effects, as well as in preparation for the next pandemics and other global health challenges.

## Data Availability

Not applicable.
